# Multivariate chemometrics as a key tool for prediction of K and Fe in a diverse German agricultural soil-set using EDXRF

**DOI:** 10.1038/s41598-019-53426-5

**Published:** 2019-11-26

**Authors:** Dominique Büchele, Madlen Chao, Markus Ostermann, Matthias Leenen, Ilko Bald

**Affiliations:** 1 Federal Institute for Materials Research and Testing (BAM), Process Analytical Technology, Richard-Willstätter-Straße 11, 12489 Berlin, Germany; 2 University of Potsdam, Institute of Chemistry - Physical Chemistry, Karl-Liebknecht-Straße 24-25, 14476 Potsdam-Golm, Germany; 3 University of Bonn, Institute of Crop Science and Resource Conservation (INRES) - Soil Science and Soil Ecology, Nussallee 13, 53115 Bonn, Germany

**Keywords:** Environmental sciences, Geochemistry, Environmental monitoring

## Abstract

Within the framework of precision agriculture, the determination of various soil properties is moving into focus, especially the demand for sensors suitable for *in-situ* measurements. Energy-dispersive X-ray fluorescence (EDXRF) can be a powerful tool for this purpose. In this study a huge diverse soil set (n = 598) from 12 different study sites in Germany was analysed with EDXRF. First, a principal component analysis (PCA) was performed to identify possible similarities among the sample set. Clustering was observed within the four texture classes clay, loam, silt and sand, as clay samples contain high and sandy soils low iron mass fractions. Furthermore, the potential of uni- and multivariate data evaluation with partial least squares regression (PLSR) was assessed for accurate determination of nutrients in German agricultural samples using two calibration sample sets. Potassium and iron were chosen for testing the performance of both models. Prediction of these nutrients in 598 German soil samples with EDXRF was more accurate using PLSR which is confirmed by a better overall averaged deviation and PLSR should therefore be preferred.

## Introduction

In recent years, precision agriculture has become increasingly important especially for targeted fertilisation due to cost reduction, reduction of environmental pollution and crop yield increase due to rising population^1–3^. Adjusted fertilisation highly depends on the requirements of the plant and knowledge of different soil properties like texture, organic and inorganic carbon content, pH and elemental composition^4–7^. However, given that the distribution of elements within a field varies widely, site-specific optimised introduction of plant essential macro and micro nutrients is required^8^. As conventional routine analysis for determination of nutrients in soils, wet digestion followed by measurement with inductively-coupled plasma optical emission spectrometry/-mass spectrometry (ICP-OES/ICP-MS) is used^9,10^. Disadvantages such as time-consuming sample preparation, possible contamination of chemical reagents and loss of volatile analytes through heating the sample solution may occure^11,12^. It has to be considered that XRF provides total contents while the plant can only absorb plant available nutrients. Especially for precision agriculture where it is helpful to combine many sensors for cultivation soils more effectively and sustainably and to improve soil functions, a combination of XRF with Raman spectroscopy could be considered. Since Raman spectroscopy identifies the compounds of the element, it is possible to estimate the plant available amount of nutrients with help of the total content. Especially for fast online measurements, a combination of both methods would be an improvement.

X-Ray fluorescence methods for detection of element specific radiation are already known as a fast, non-destructive multi-element spectroscopic technique and have been widely applied in a variety of fields, such as agricultural^13^ and geological^14^ applications or environmental^12,15^ monitoring. EDXRF has already been used for the analysis of trace elements as well as major and minor nutrients^16^ in soils from different countries such as contaminated soils in the UK^17^, clayey and sandy soils from Denmark^18^ or heavy trace elements in soils from Australia^19^ with univariate calibration. Univariate data evaluation uses only one variable of the spectra and doesn´t take any causes or relationships into account. Regarding the complex soil matrix, chemometric methods based on mathematical models considering the entire spectral information and therefore more variables, offer another way to traditional univariate regression and have already been used for optical spectroscopic analysis^20,21^. Multivariate data analysis is especially suitable for large data sets as they occur during sampling of arable land. PLSR as one chemometric approach provides the opportunity to accomplish a multivariate regression relationship and has also the ability to overcome matrix effects^11^.

Recent papers have shown that multivariate data analysis can also be applied to XRF for example for the classification of different types of Argentine soils^10^, determination of heavy metal concentrations^22^ or the element content in soils from India^8^, Northern Ireland^23^ or France^24^. For calibration with chemometric tools, Kaniu *et al*. has tested the performance of PLSR and artificial neural network (ANN) in combination with energy-dispersive X-ray fluorescence and scattering spectroscopy for soil quality assessment in Kenja^11,25,26^. It was also possible to predict the sand or clay content with chemometric tools such as PLSR^27,28^. Prior studies have treated soils from different countries all over the world with small sample sizes or sample sets from one or two study sites, but less work has been carried out for studying such a large selection of German agricultural soils as presented in this paper.

From 12 different study sites all across Germany, 598 field samples were collected with widely varying texture. First, PCA of the spectra was conducted for clustering the samples and to identify similarities within the sample set. The second aim of this study was to test the potential of two different calibration methods in combination with EDXRF for accurate and reliable determination of important analytes in German agricultural soils. As a case study, this paper is focused on K and Fe. In the plant, potassium is responsible for the adjustment of osmotic pressure and the regulation of the water balance, while iron is extremely important as a component for chlorophyll and proteins and activates different enzymes for photosynthesis and energy metabolism^29^.

Traditional univariate analysis and multivariate partial least squares regression were applied as method of choice. Univariate calibration is simple especially with regard to data handling while PLSR often leads to better prediction ability, as the attained robustness of the spectra is higher^30^. To receive a matrix-specific calibration either certified reference materials (CRM) or a subset of the German field samples were used. For independent validation purpose the concentration of K and Fe in the sample set (n = 598) was predicted and compared by means of standard methods such as ICP-OES and WDXRF. The capability of both regression methods in combination with the two calibration sample sets was studied by comparing the overall averaged deviation of the prediction of unknown samples as a key parameter.

## Results and Discussion

### Principal component analysis for classification of German soil sample

Principal component analysis can be applied as a qualitative classification approach and for detection of outliers within repetition measurements of the same sample by reducing the dimensionality of the data set. This leads to separation of information arising from noise and to identification of a few influential and statistically relevant variables^31^. To achieve this, new latent variables are formed as linear combination from the original variables, so a transformation of the data in a new orthogonal coordinate system with uncorrelated so-called principal components (PC) is conducted. The data are split according to their major variance, making the relevant similarities and differences in the data set visible in a score plot^8,32,33^.

The original data set consists of a matrix with 15 rows (CRM) and 2048 columns (fluorescence intensities) from the corresponding energy channels −0.02 to 25.68 keV. Parts of the spectra that didn´t contain any peaks were omitted for clarity and for better interpretation of the loading plot. Looking at the PCA score and loading plot, no differences were observed between the full and the reduced (1.00–16.49 keV) spectra. CRM NCS DC87104 and NIST 2710 were excluded from the PCA prior to modelling, so only 13 CRM were used (see Supplementary Information Fig. S1). When performing PCA the software The Unscrambler® X automatically mean-centered the data and auto-scaling was done. Furthermore, non-linear iterative partial least squares (NIPALS) was used as algorithm and a cross-validation of the CRM was conducted. The spectrum in the region between 2.70 and 3.18 keV, that is related to the Ag-L_α_ peak of the X-ray tube, showed peak fluctuations between repeat measurements, and hence, this area was down-weighted automatically by the software for calculation to prevent influences on the PCA.

**Figure 1:**
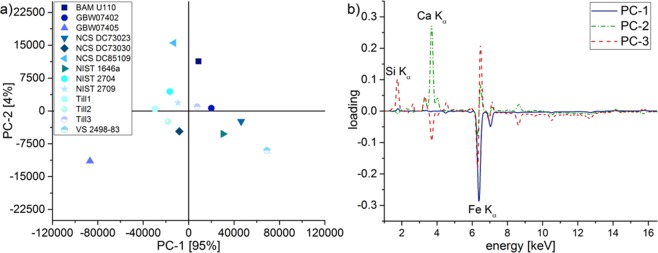
PCA score plot (**a**) of EDXRF soil data for the first two principal components PC-1 and PC-2 of 13 certified reference materials with a total variance of 99%. Corresponding loading plot (**b**) of the first three principal components for CRM. Loadings are plotted against the energy [keV]. PC-1 corresponds to iron, PC-2 to calcium and PC-3 to silicon.

First, a PCA was implemented using the spectra of the 13 highly diverse CRM. In the second step, the projection of the 409 arable soil samples (409 × 1234 matrix) into this PCA took place. For this, the software compares the spectra of the samples with those of the CRM to classify them into the PCA. This procedure facilitates the integration of further samples in perspective to later *in-situ* measurements on arable land. In Fig. 1, the score plot of 13 CRM based on PC-1 and PC-2, describing a total variance of 99%, is shown. The data points are all within the 95% confidence level for T² (see Supplementary Information Fig. S1).

The relation between the original and the new variables is expressed through the so-called loadings. A loading plot shows which properties of the samples, in this case elements, define the principal components and thus, which elements in the soil matrix show the greatest variance within the data set^12^. The first principal component should only be related to relevant fluorescence peaks allowing the sorting of the samples^34^. Looking at the corresponding loading plot in Fig. 1, main clustering was based on the fluorescence intensity of iron. A strong peak with a negative loading value arising from the Fe K_α_ peak characterises PC-1 and leads to 95% of the total variance. In conclusion, a sample with a high concentration of iron corresponds to a point located on the left side in the negative area of the horizontal PC-1 axes in the score plot. This agrees with the results given that CRM GBW07405 with the highest iron content of 8.83 wt-% is farthest to the left while Till3 with 2.74 wt-% is close to a score of 0 and VS 2498-83 with 0.69 wt-% is the sample with the highest PC-1 score. Similar correlation between PC-1 and Fe with a high variance was proposed by Kaniu *et al*., who used energy-dispersive X-ray fluorescence and scattering spectroscopy for assessment of soil quality. Moreover, soils were clustered according to their soil type and not to their texture^25,35^. The second PC is dominated by calcium with a peak in the positive area of the loading plot and a residual variance of 4%. Splitting of the CRM along the vertical axis of PC-2 in the middle mass fraction range works only to a limited extent due to the low variance. Samples with high Ca content like NCS DC85109 are in the positive area and GBW07405 with a low content in the negative area with the lowest score value.

**Figure 2:**
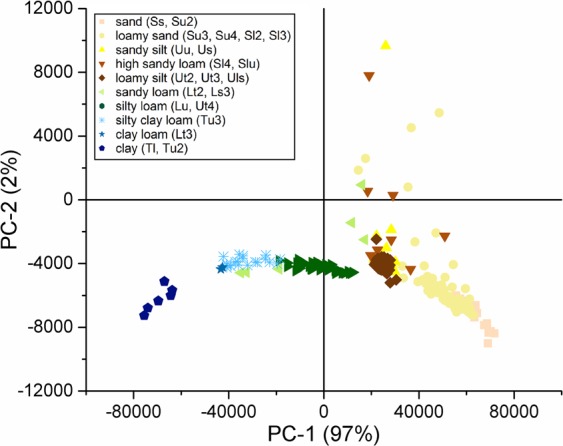
PCA score plot, projection of the EDXRF German agricultural soil data for the first two principal components PC-1 and PC-2 of 13 certified reference materials.

Figure 2 shows the score plot of 409 projected soil samples from 12 different study sites in Germany. All PC´s for the projected samples account for 99% of the total variance whereas PC-1 dominates around 97%. Regarding the results for PCA of CRM, a distinction between the different German soils is possible due to their variation in PC-1. The separation of the samples in the score plot fits to reference values of ICP-OES and WDXRF with respect to the iron and calcium content.

In the score plot, all samples were labelled with colours by their texture according to their classification by the German VDLUFA. Superordinate classes of soils in consideration to VDLUFA texture are sand, silt, loam and clay with different subdivisions^36^. The subdivisions of the soil types differ by their sand, silt, loam and clay content. As shown in Fig. 2, samples with the same colour and therefore the same texture cluster together. The lowest scores were mostly associated with clay samples while sandy samples offered the highest score for PC-1. Given that the solubility of Fe oxides is extremely low they are especially located in the clay fraction^27,37,38^. In accordance with this, the splitting of the soils corresponds with the results of iron content. Similar correlations between iron and clay content were also observed in soils from the USA and Angouran Area though no comparison and classification of texture was done^1,27^. Clay samples on the left-hand side contain the highest amount of iron (>4 wt-%). Sandy samples are in the right area of PC-1 with a low iron content of <1.1 wt-%. Samples of sandy loam are split into two groups. One group is closer to silty clay loam (Tu3) while the other group is located near the silt samples. This can be explained by the different subdivisions of sandy loam: according to VDLUFA the subdivisions are St3, Ls4, Ls3, Ls2 with the same clay content, while Lt2 has a higher clay content. The sandy loam samples used in this study only belong to the group Ls4 and Lt2. The samples in the upper part belong to Ls4 while the other three samples are Lt2. Lt2 has a higher clay content (25–35%) than Ls4 (17–25%), so considering the results, also a higher Fe content. Besides this, their calcium content (>0.95 wt-%) is higher than the one for Lt2 (<0.5 wt-%). The silty samples such as sandy silt, high sandy silt and loamy silt are grouped together. These samples have a similar iron content, so that a separation of the individual subdivisions is not possible by PCA. All outliers in the upper quartile of the score plot have a high mass fraction of Ca compared to the other samples in this subdivision. Taking this fact into account a classification of these soils is possible on basis of the other samples within the same field.

Classification of subordinate texture using PCA as chemometric tool combined with this EDXRF set-up was possible for a variety of 409 German arable soils. Given that all samples are located within the 95% confidence level they are well described by the model. On basis of the iron content a separation of the different soils by their texture according to VDLUFA^36^ on the PC-1 axes was possible. Clay containing samples have the highest amount of iron while the iron content decreases with increasing silt and sand ratio. With view to later field-application a division of the texture of agricultural soils from Germany is possible. Knowing the texture is especially important given that it affects the crop growth environment for example for water-holding capacity^39^. Further development should be done by classification of more field samples with known texture, especially clay samples, from different sites in Germany.

### Univariate data analysis for determination of nutrients

**Figure 3:**
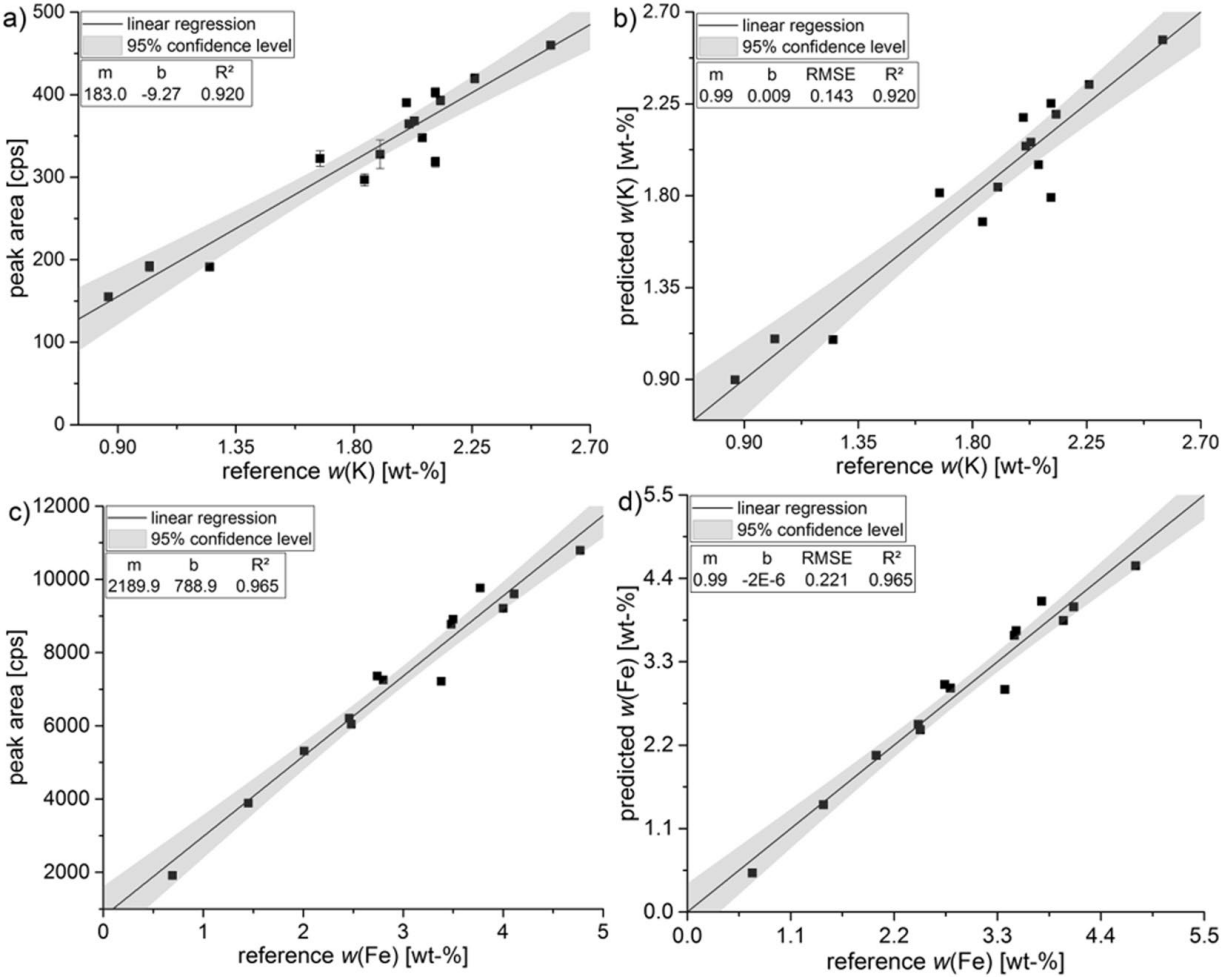
Univariate calibration model for potassium (**a,b**) and iron (**c,d**) based on 15 CRM. The net peak area of the characteristic fluorescence peak [cps] was fitted against the mass fraction in the CRM [wt-%] (**a,c**). The error bars represent the standard deviation of five measurements. For comparison purpose of the univariate calibration model: predicted values [wt-%] were fitted against the reference values [wt-%] (**b,d**).

The calibration models obtained by univariate data analysis are shown in Fig. 3a,c. Averaged net peak area of the characteristic fluorescence peak of the analyte is fitted against the known mass fraction in the 15 CRM. A linear regression is obtained with the equation y = *m* * x + *b*, where *m* denotes to the slope or sensitivity and *b* to the intercept. A strong correlation is reflected by the coefficient of determination (*R²*), which should be almost equal to 1, and the root mean square error (*RMSE*), which should be close to 0^40^. *R²* for K and Fe (0.920; 0.965) as well as the small error bars demonstrate a good linear relationship where just a few mean values were not within the 95% confidence level. For later comparison of the RMSE with the multivariate one, the univariate predicted values of K and Fe were fitted against the certified reference values (see Fig. 3b,d).

For validation of the calibration model and determination of the accuracy, the CRM IAEA Soil-5 was analysed and the mass content was calculated. It is important to note, that this reference material was not used to generate the linear fit. For the univariate calibration model with CRM a mass content of 1.90 ± 0.03 wt-% for K and 4.29 ± 0.05 wt-% for Fe was predicted (see Table 1). These values are in accordance to the certified values (K: 1.86 ± 0.15 wt-%; Fe: 4.45 ± 0.19 wt-%) which is confirmed by a good recovery rate of 102.2% and 96.4%. Through validation, the quality of the obtained calibration model could be monitored.

**Table 1:** Comparison of certified mass content [wt-%] compared to predicted mass content [wt-%] of potassium and iron with either univariate or multivariate calibration models with 15 CRM and a subset of 41 German agricultural soil samples.

Element	Calibration with	Certified mass content/wt-%	Predicted mass content/wt-%	
			univariate	multivariate
K	15 CRM	1.86 ± 0.15	1.90 ± 0.03	1.88 ± 0.06
	41 agricultural soil samples		1.89 ± 0.04	1.90 ± 0.05
Fe	15 CRM	4.45 ± 0.19	4.29 ± 0.05	4.28 ± 0.08
	41 agricultural soil samples		4.17 ± 0.06	4.22 ± 0.08

**Figure 4:**
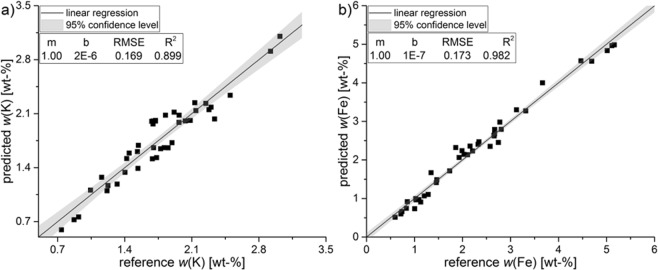
Predicted values [wt-%] of potassium (**a**) and iron (**b**) using univariate analysis with 41 German agricultural soils fitted against the reference values of WDXRF [wt-%].

A calibration was also done by a selected subset of German soil samples (Supplementary Information Tables S8, S9). The samples were chosen randomly by looking at their element mass content to receive a wide linear range (*n* = 41). From every study side at least two samples were used. In Fig. 4, the predicted values were fitted against the reference values of WDXRF for the selected samples. A high degree of linearity was maintained for both analytes (*R²* = 0.899 K, 0.982 Fe). For K the RMSE increases (0.143 vs. 0.169) compared to the calibration with CRM, while the opposite effect is observed for Fe (0.221 vs. 0.173). Validation of the univariate calibration with agricultural soils achieved a recovery of 101.6% for K and 93.6% for Fe in IAEA Soil-5.

### Partial least squares regression for determination of nutrients

The model was created by the Y-variables (mass fraction of the analyte) as a function of the X-variables (intensity of the fluorescence)^25,41^. In this work the used dataset for calibration with 15 CRM was a 75 × 1234 matrix and for prediction of German agricultural soils a 2990 × 1234 matrix (598 samples, 5 repetitive measurements). As already established for PCA, same parts of the spectra with no spectral information were removed. Different methods for data treatment of the raw spectra including background correction, smoothing and Savitzky-Golay derivation were applied to obtain the best cross-validation model (lowest RMSEC and RMSEV) and the best prediction ability. Savitzky-Golay derivation (polynomial order: 2, smoothing points: 3) for K and a linear baseline correction for Fe as data pre-treatment were selected. Due to varying matrix composition of each soil sample, the region of interest was carefully selected (K: 3.19–3.48 keV; Fe: 6.1–7.3 keV) and fluorescence signals not related to either potassium or iron were down-weighted with the software The Unscrambler® X^21^. For pre-processing, mean-centering was done automatically by the software. Kernel as algorithm was used and for validation of the PLSR model leave-one-out cross-validation was chosen.

**Figure 5:**
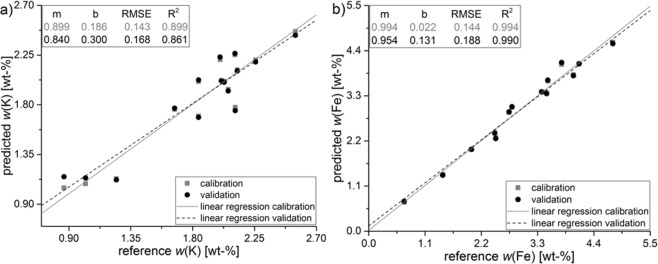
Partial least squares regression of 15 CRM for K (**a**) and Fe (**b**) using Savitzky-Golay derivation, linear baseline correction as data pre-treatment, Kernel as algorithm and leave-one-out cross-validation.

Figure 5 shows the PLSR of 15 CRM as calibration-set. The linear regression for calibration (line) and validation (dotted line) differ little from each other, whereupon the validation regression is located within the 95% confidence level of the calibration. Predicted Y-variables (mass content) are highly correlated with the reference Y-variables in a linear relationship. *R²* for calibration and validation are close to each other and account 0.899/0.861 for K and 0.994/0.990 for Fe, while RMSE is 0.143 and 0.144 for calibration. The linearity of the model is given between 0.8–2.6 wt-% for potassium and 0.6–5.4 wt-% for iron in soil samples using this EDXRF set-up combined with PLSR.

For external validation of the PLSR the analyte contents in CRM IAEA Soil-5 were predicted with a value of 1.88 ± 0.06 wt-% for K and 4.28 ± 0.08 wt-% for Fe. Under consideration of the error margin, the predicted mass fractions are in good agreement with the certified values (see Table 1). With PLSR, a recovery of 101.1% and 96.2% was achieved indicating that multivariate regression leads to an accurate and robust quantification of potassium and iron in German agricultural soil samples.

**Figure 6:**
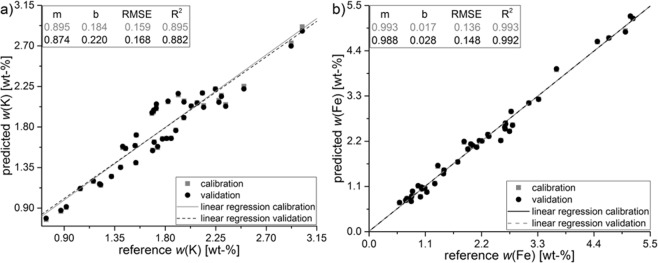
Partial least squares regression with 41 German agricultural soils for K (**a**) and Fe (**b**) using Savitzky-Golay derivation, linear baseline correction as data pre-treatment, Kernel as algorithm and leave-one-out cross-validation.

In addition, PLSR was carried out with the same selected 41 German soil samples (205 × 1234 matrix) as already mentioned in the section before (see Fig. 6). Pre-treatment of the dataset and region of interest were the same as for calibration with CRM.

Predicted values and reference values (WDXRF) for both analytes are closely correlated for PLSR of agricultural soils. Especially calibration and validation for potassium are in better accordance now. For Fe the linear regression is nearly the same as for CRM, while RMSE is better for calibration with soils (0.144 vs. 0.136). 102.2% and 96.2% recovery rate were received when predicting K and Fe in IAEA Soil-5.

### Comparison of univariate and multivariate calibration strategies for German soils

The performance of traditional uni- and multivariate matrix-specific calibration with CRM as well as a selection of German agricultural soils was evaluated to determine nutrients, in this study K and Fe, in German agricultural soils considering statistical parameters. As a key parameter, the overall averaged deviation of the predicted values in 598 German soil samples was applied indicating that the used German data set is described well by the models.

Comparing *R²* for uni- and multivariate regression with CRM, a better correlation (0.965 vs. 0.994) was achieved for iron when using PLSR, while for potassium *R²* was better for univariate calibration (0.920 vs. 0.899). Furthermore, the multivariate calibration leads to a similar or lower RMSE (K: 0.143 vs. 0.143; Fe: 0.221 vs. 0.144) and a better recovery rate (K: 102.2 vs. 101.1%; Fe: 104.9 vs. 96.2%) in IAEA Soil-5 for both elements, respectively (Table 1).

To receive an even more customised matrix-specific calibration, a linear regression with a subset of German soil samples was conducted. It was expected to achieve a more reliable model and to increase prediction ability and thus the accuracy. As already observed for the calibration with CRM, the multivariate calibration leads to a better *R²* for iron (0.982 vs. 0.993), a similar *R²* for potassium (0.899 vs. 0.895) and lower RMSE (K: 0.169 vs. 0.159; Fe: 0.173 vs. 0.136) for both analytes when using German field samples as calibration set. A comparison between the 15 CRM and the 41 subset soils shows, that the calibration varies only slightly. Basically, RMSE for K is increasing and decreasing for Fe.

For all 598 agricultural soils K and Fe content was determined with both evaluation methods as independent validation. To compare the predicted values of the two approaches, reference values for the sample set were obtained with WDXRF and ICP-OES. The number of samples with absolute deviations <5%, 5–10%, 10–15%, 15–20% and >20% was added together for better comparability of the values. The predicted mass fraction in the sample set ranges from 0.6 to 3.1 wt-% for potassium and from 0.6 to 5.5 wt-% for iron and reflects the real element content in German agricultural soils, which can vary appreciable. It should be considered, that the mass content of both analytes in the calibration set covers a wide range of mass fractions, so linear dynamic range for all linear regressions should be sufficient.

As a key parameter, the absolute deviations between predicted and reference WDXRF values were averaged over all 598 samples to determine the method with the lowest averaged deviation and therefore with the best accuracy and precision^24^. Since the models are slightly different (*R*^2^ and RMSE) and the recovery rate is very similar, this average value is a good option to compare the models with each other. If the averaged deviation is small, the bias between predicted and reference values is low and a high accuracy is achieved. The value is representative, as it reflects the deviations of a huge sample set. In Table 2 the descriptive statistics for the prediction of K and Fe in the sample set is listed and for a detailed evaluation, the absolute deviations were divided in different deviation areas.

**Table 2:** Number of samples with absolute deviations summarized in deviation areas and the descriptive statistics of those deviations for the prediction of K and Fe content in 598 German agricultural soils using either univariate (uni) regression or PLSR (multi) with 15 CRM or with 41 German soil samples for calibration.

		Average deviation/%	Median/%	MAE^a^/wt-%	Number of samples with absolute deviations^b^				
					<5%	5–10%	10–15%	15–20%	>20%
**Calibration with 15 CRM**									
K	uni	8.45	7.28	0.15	210	170	108	96	14
	multi	7.19	6.96	0.13	261	168	143	23	3
Fe	uni	11.79	11.37	0.22	110	110	244	71	63
	multi	4.40	3.50	0.08	403	145	37	12	1
**Calibration with 41 agricultural soil samples**									
K	uni	9.16	8.22	0.16	200	159	95	113	31
	multi	8.03	6.74	0.14	227	163	119	82	7
Fe	uni	7.74	7.50	0.15	185	252	120	24	17
	multi	4.40	3.50	0.08	403	145	37	12	1

^a^MAE = Mean absolute error (see Section Material and Methods). ^b^Number of samples with absolute deviations <5%, 5–10%, 10–15%, 15–20% and >20%.

For both analytes, PLSR with 15 CRM leads to the lowest averaged deviation with 7.19% for K, 4.4% for Fe while univariate calibration yields in 8.45% and 11.79%. Particularly, for PLSR with CRM more samples were predicted with derivations <10% while less samples drift more than 15% (K: 26 vs. 110; Fe: 13 vs. 134). This can be confirmed by the mean absolute error (MAE) of multi- and univariate prediction: 0.13 vs. 0.15 wt-% for K and 0.08 vs. 0.22 wt-% for Fe. The mean coefficient of variation for all predicted values was below 3% for K and Fe indicating a high repeatability and accuracy at a fast scanning-time of 60 s.

As mentioned earlier, the calibration of CRM and German agricultural soils are quite similar for both uni- and multivariate regression, still using CRM leads to a better prediction ability. For example, the averaged deviation for potassium increases from 8.45 to 9.16% for univariate and from 7.19 to 8.03% for multivariate regression. On the contrary, using CRM for univariate calibration yields in a higher averaged deviation (11.79 vs. 7.74%) for iron compared to calibration with German soils, while there is no impact between the multivariate calibrations (4.40 vs. 4.40%). The CRM have the advantage that they are better characterised compared to the real soil samples. They are examined in round robin tests by several laboratories and the certified value is statistically calculated from different values. In addition, the CRM are grounded to small grain sizes. The real soil samples were also grounded but their grain sizes are much bigger (<500 µm). That can be the reason for this bias.

Robust and reliable calibration models were achieved for all regression methods with high *R²* and low RMSE. External validation with IAEA Soil-5 showed that the obtained calibration curves performed very well indicated by a good recovery rate between 94–105%. The averaged deviation for all 598 soil samples was below 12% for uni- and multivariate calibration with both sample sets (CRM and selected subset). Thus, univariate regression can also be used to determine nutrients in German arable soils with focus on the prediction ability, however a multivariate evaluation results in a significantly higher accuracy and precision. Moreover, the data handling, especially when using the reduced spectra with less data points is easy to manage and as fast as the univariate data treatment. The advantage of the multivariate PLSR is that the entire spectrum is taken into account for modelling the linear regression. As a result, PLSR is capable of compensating matrix effects. Particularly, when changes in the peak shape occur then the PLSR considers them. In this case, both reasons lead to a higher accuracy and precision compared to the univariate data evaluation.

Despite the challenges of analysing light elements such as K with XRF, the prediction ability and the accuracy for K are comparable to these of Fe, which is easier to detect due to better resolution and a higher atomic weight. In addition to the difficulties in analysing light elements by means of poorer resolution and lower fluorescence yield, the prediction ability can be increased by calibration with CRM instead of a selection of German agricultural soils.

Hence, EDXRF as sensor can be used to achieve a fast, accurate and reliable prediction of analytes in unknown German agricultural soils and analyser performance can be improved by coupling with multivariate data evaluation using PLSR instead of univariate data analysis. Further work is dedicated to convert the information collected for lab measurements of German soils with prepared samples to *in-situ* soil measurements considering particle size and soil moisture^15,27^.

## Materials and Methods

### Sampling and sample preparation of the German soil sample set

In this investigation, 598 different soil samples with varying levels of element content (0.64–3.10 wt-% for K; 0.59–5.4 wt-% for Fe) and texture extracted from arable land were used. Soil samples and information about location, horizon and soil texture were provided by S. Pätzold and M. Leenen, Institute of Crop Science and Resource Conservation (INRES), Soil Science and Soil Ecology, University of Bonn (Germany), A. Mizgirev, Institute of Agricultural and Nutrition Sciences, Martin-Luther University of Halle/Wittenberg (Germany), R. Gebbers, Department of Engineering for Crop Production, Leibniz Institute for Agricultural Engineering and Bioeconomy (ATB, Potsdam, Germany) and E. Wallor, Institute of Landscape Systems Analysis, Leibnitz Institute for Agricultural Landscape Research (ZALF, Muencheberg, Germany)^37^. The samples were collected in the field from 12 different study sites in Germany. An analysis of the texture was done by other laboratories or the project partners themselves. Extended information about the sample set are listed in the Supplementary Information (Table S1).

All samples were air dried at room temperature and sieved to grain sizes smaller than 2 mm with a 2 mm-mesh stainless steel sieve. Samples (A, G, Zalf, Goerzig) were homogenised through the cross-riffling method using the rotary cone sample divider from Fritsch, model Laborette 27 (Germany). Each sample was mixed by grinding in a Spex Mixer/Mill from Spex Industries Inc. (USA) to suitable grain size for preparation of homogenous pellets. Pressed pellets were prepared with a force of 10 kN for 10 s in a hydraulic press machine, Model HTB 40 from Herzog (Germany). 7 g of each sample was placed in an aluminium cup and pressed without additional binder to a 32 mm diameter pellet.

Relative methods such as XRF require standard materials with known elemental composition for quantitative evaluation of the spectra. The matrix of standard materials should be as similar as possible to the unknown samples for matrix-specific calibration and to diminish the absorption-enhancement effects^10,20,40^. For calibration of the sensor, 15 CRM from different manufactures were used. All CRM offer different elemental compositions (0.86–2.55 wt-% for K; 0.69–5.5 wt-% for Fe): BAM U110; GBW07402; GBW07405; NCS DC73023; NCS DC73030; NCS DC85109; NCS DC87104; NIST 1646a; NIST 2704; NIST 2709; NIST 2710; TILL1-3; VS 2498-83. Details are listed in the Supplementary Information Tables S2, 3. IAEA Soil-5 has served as external validation sample for both regression methods. The standard soil LUFA 2.2 and 2.3 with certified texture was only used for PCA with lack of total values.

### Experimental setup for EDXRF analysis

X-ray fluorescence spectra were obtained by using a non-commercial energy-dispersive spectrometer constructed by the Institute for Applied Photonics e.V. (Germany). The instrument is equipped with an Ag-target X-ray tube with a maximum power of 30 W. A peltier cooled 25 mm^2^ thick silicon drift detector (Amptek SSD-123×) with a resolution of about 145 eV for Mn-K_α_ X-ray line at 5.9 keV was used as detection unit. The built-in collimator is 0.5 mm thick with a cross-diameter of 5 mm. Incident and take-off angles were 42°. The distance between tube and sample was 8 cm, between sample and detector 2.5 cm. As medium between tube, detector and sample helium was chosen for detection of light elements. Weighing less than 2 kg this setup can be used for later on-site measurements.

### Evaluation and analysis of X-ray spectra

Spectra were acquired using a voltage of 29.7 kV, a current of 0.49 mA and a spectral acquisition time of 60 s. Experimental parameters were optimised for the wide range of elements in soil in the region of interest from K–Zn with regard to later *in-situ* application on the field. High signal-to-noise ratios could be achieved at the respective current, voltage and acquisition time, hence, a compromise between EDXRF sensitivity and analytical speed was chosen. A representative spectrum of an arable soil sample is shown in the Supplementary Information Fig. S4. With this setup, K_α_ of potassium and K_α_ as well as K_β_ of iron were detectable at an energy of 3.29, 6.31 and 7.06 keV. The iron peaks are well resolved and therefore good to analyse while potassium has a spectral overlap with Ag-L_α_ from the X-ray tube that needs appropriate data pre-treatment.

The following software was used: Origin^®^ 2016G (OriginLab, USA) and the instrument software Elbrus (IAP, Germany) for univariate evaluation, The Unscrambler® X Version 10.5 (Camo, Norway) for modelling the PCA and PLSR.

Chemical and physical properties differ from one soil to another soil. In particular, the composition of major and minor nutrients is significantly diverse as well as the grain size distribution^33,34,39^. Particle sizes can influence the quantification results especially for light elements as they are present in soils^18^. The German field samples were grounded to obtain a homogenous surface but still inhomogeneity of the pellets could be seen by eyes at parts of the sample. For this reason and to avoid matrix effects, a matrix-specific calibration was carried out using either CRM or a selected subset of the German agricultural soils. In the case of XRF, matrix effects are the results of different mass fractions of interfering elements, which may have an impact on the detected X-ray intensity of the analyte. Response of the intensity to the mass content is not linear anymore, so X-ray absorption or enhancement effects may occur^30,42^.

The obtained X-ray spectra for CRM and soil samples were smoothed (number of smooths: 2), background corrected, and each evaluated peak was automatically fitted with a Gaussian function by the instrument’s software. The calculated net peak areas for the characteristic element lines were used for evaluation with univariate analysis whereas for multivariate analysis the raw spectra were pre-treated prior to modelling. Each pellet was measured at five different positions to consider inhomogeneity of the soil samples and to reduce statistical error^9^. For univariate data analysis the peak areas were averaged while for multivariate data analysis the software is able to average the five spectra to one spectrum prior to modelling.

Considering statistical parameter, coefficient of determination and root mean square error, the potential of univariate regression and chemometric PLSR is discussed. Prediction ability was evaluated by calculating the overall averaged deviation and the mean absolute error of prediction (MAE) as follows^24,37^:

1 $$Averaged\,deviation=\frac{1}{n}\mathop{\sum }\limits_{i=1}^{n}\,[\frac{|{y}_{i,Ref}-{y}_{i,Pred}|}{{y}_{i,Ref}}]\,\ast \,100$$

2 $$MAE=\frac{1}{n}\,\mathop{\sum }\limits_{i=1}^{n}\,|{y}_{i,Ref}-{y}_{i,Pred}|$$

where *n* is the number of German soil samples (*n* = 598), ${y}_{Ref}$ is the reference value of the analyte with WDXRF and ${y}_{Pred}$ is the predicted value of the analyte using either uni- or multivariate calibration with EDXRF.

Instrument stability was controlled using CRM GBW07405 and agricultural soil Zalf_010. Replicate measurements at the same position (*n* = 5) and measurements at different positions of the pellets (*n* = 5) were conducted to calculate the precision^43^. Both, spatial heterogeneity and precision are reflected by the relative standard deviation (RSD) represented as error bars. The RSD for potassium and iron net peak area was below 8% and 10%, while the mean coefficient of variation for all 598 soil samples was below 3% indicating that the repeatability is very well for both analytes.

### Reference values of the german sample set

For ICP-OES, prior microwave assisted HF-digestion was performed with an Anton Paar Multiwave 3000 (Austria). The used ICP-OES instrument was an Agilent 5100 with pneumatic nebulisation. Due to time-consuming sample preparation, a selection of 70 representative German soil samples were measured. WDXRF measurements were done with a MagiX Pro from Panalytical (The Netherlands), equipped with a water-cooled Rh-tube. Regarding the high amounts of sample and the need for binder because of the upside-down instrument set-up, no pressed pellets were used instead the 598 soil samples were placed in a X-ray sample cup covered with a 6 µm X-ray Mylar^®^ foil. WDXRF was calibrated with the same 15 CRM listed above and the calibration was done automatically by the software SuperQ 5.1B. Helium was used as atmosphere. Further details are listed in the Supplementary Information (Tables S5–S7, Fig. S3).

## Supplementary information

Multivariate chemometrics as a key tool for prediction of K and Fe in a diverse German agricultural soil-set using EDXRF

## Data Availability

The data generated during the current study are available from the corresponding author on reasonable request.
