# Supplementary material for: Multivariate chemometrics as a key tool for prediction of K and Fe in a diverse German agricultural soil-set using EDXRF
Source: Sci Rep. 2019 Nov 26;9:17588. doi: 10.1038/s41598-019-53426-5 (PMC6879603; doi:10.1038/s41598-019-53426-5)
Supplement: Supplementary file 1 — Multivariate chemometrics as a key tool for prediction of K and Fe in a diverse German agricultural soil-set using EDXRF [file 41598_2019_53426_MOESM1_ESM.pdf]

Multivariate chemometrics as a key tool for prediction of K and Fe in a diverse German agricultural soil-set using EDXRF

Dominique Büchele,*[,1,2] Madlen Chao,[1,2] Markus Ostermann,[1] Matthias Leenen,[3] Ilko Bald[1,2]

[1]Federal Institute for Materials Research and Testing (BAM), Process Analytical Technology, Richard-Willstätter-Straße 11, 12489 Berlin, Germany. [2]University Potsdam, Institute of Chemistry - Physical Chemistry, Karl-Liebknecht-Straße 24-25, 14476 Potsdam-Golm, Germany. [3]University of Bonn, Institute of Crop Science and Resource Conservation (INRES) - Soil Science and Soil Ecology, Nussallee 13, 53115 Bonn, Germany. Correspondence and requests for materials should be addresses to D.B. (email: dbuechele@uni-potsdam.de)

Supplementary Information

Table S 1: *Additional information about the German agricultural soil samples in this study.*

Study site	Sample	Soil type	Horizon [cm]
Marquardt*	A1	sand (Su2)	0 – 25
Marquardt*	A2	sand (Su2)	25 – 50
Marquardt*	A3	sand (Su2)	70 – 95
Ascheberg, Bonn	G1	loamy sand (Sl3)	0 – 30
Ascheberg, Bonn	G2	clay (Tl)	0 – 30
Campus Klein-Altendorf, Bonn	G3	loamy silt (Ut3)	0 – 30
Campus Klein-Altendorf, Bonn	G4	sandy silt (Uu)	130 – 150
An der Chaussee, Gut Wilmersdorf⁺	ATB	sand (Su2), loamy sand (Su3, Su4, Sl2, Sl3), high sandy silt (Sl4), sandy loam (Ls4)	0 – 30
Kohlgarten, Gut Wilmersdorf⁺	ATB	loamy sand (Su3, Su4, Sl2, Sl3), high sandy silt (Sl4),	0 – 30
Rotes Meer, Gut Wilmersdorf⁺	ATB	sand (Su2), loamy sand (Su3, Su4, Sl2, Sl3)	0 – 30
Waschpuhl, Gut Wilmersdorf⁺	ATB	sand (Su2), loamy sand (Su3, Su4, Sl2, Sl3), high sandy silt (Sl4), sandy loam (Ls4)	0 – 30
Goerzig[#]	Goerzig	sandy silt (Us), loamy sand (Su3), loamy silt (Ut2)	0 – 30
Zalf, Muencheberg	Zalf	sand (Su2), loamy sand (Su3, Sl2)	0 – 25
Grafschaft Boelingen	UBR	sandy silt (Lt2), silty loam (Lu, Ut4), silty clay loam (Tu3), clay loam (Lt3), clay (Tu2)	0 – 30
Various soil samples with unknown site	Tex, UB	Sand (Ss), loamy sand (St2), sandy silt (Us), loamy silt (Uls, Ut2, Ut3), sandy loam (St3, Ls3, Ls2, Lt2), silty loam (Lu, Ut4), loam (Lts), silty clay loam (Tu3), clay loam (Lt3), clay (Tu2, Tl, Tt)	n.A.

* Geographical position: Northeast Germany, experimental station of the Leibniz Institute for Agricultural Engineering and Bioeconomy (ATB), coordinates: 52.465542°;12.960594°

+ Geographical position: Northeast Germany, Gut Wilmersdorf, coordinates: 53,107635°; 13,91506°
# Geographical position: Middle east Germany, Goerzig, coordinates: 51,66386°; 12,00239°

Table S 2: *K, Ca and Fe mass content, water content (H_2O), loss of ignition (LOI) in [wt-%] and texture of the certified reference materials.*

CRM	K	Ca	Fe	LOI	H_2O	Soil type
**BAM U110**	1.900*	4.100*	2.800*	13.300	2.700	sandy silt (Us)
**Till1**	1.843	1.944	4.770	5.298	1.325	–
**Till2**	2.548	0.908	3.770	5.686	1.338	–
**Till3**	2.009	1.880	2.742	3.020	1.007	–
**NCS DC85109**	2.258	5.668	4.001	5.442	2.720	–
**NCS DC87104**	1.669	6.482	2.483	4.348	0.669	–
**NCS DC73023**	2.125	1.308	1.448	1.312	0.328	–
**NCS DC73030**	2.059	0.243	3.476	4.714	1.347	–
**NIST 1646a**	0.864	0.519	2.008	2.303	0.329	–
**NIST 2704**	2.000	2.600	4.110	6.226	0.778	–
**NIST 2709**	2.030	1.890	3.500	5.172	3.017	–
**NIST 2710**	2.110	1.250	3.380	7.950	2.510	–
**GBW07402**	2.108	1.687	2.462	3.716	0.676	–
**GBW07405**	1.245	0.068	8.826	7.333	2.333	–
**VS 2498-83**	1.021	0.193	0.692	1.350	0.330	sand (Su2)
**IAEA Soil-5**	1.856	2.200*	4.454	3.998	1.380	–
**LUFA 2.2**	–	–	–	–	–	sandy loam (Sl3)
**LUFA 2.3**	–	–	–	–	–	loamy sand (Su3)

* not certified values

Table S 3: *Institute and sample site for all CRM.*

CRM	Institute	Site
BAM U110	*Federal Institute of Material Research and Testing*	Berlin, Germany
TILL1, TILL2, TILL3	*Canadian Centre for Mineral and Energy Technology*	Ottawa, Canada
NCS DC73023, NCS DC73030, NCS DC85109, NCS DC87104	*National Analysis Centre for Iron and Steel*	Beijing, China
NIST 1646a, NIST 2704, NIST 2709, NIST 2710	*National Institute of Standards and Technology*	Gaithersburg, USA
GBW07402, GBW07405	*National Research Centre for Certified Reference Materials*	Beijing, China
VS 2498-83	*ICRM-Centre*	Moscow, Russia
IAEA Soil-5	*International Atomic Energy Agency*	Vienna, Austria
LUFA 2.2, LUFA 2.3	*Landwirtschaftliche Untersuchungs- und Forschungsanstalt Speyer*	Speyer, Germany

Table S 4: *Different soil textures with their subdivisions according to the German classification system of soils: VDLUFA.*

	Group of soil type	Subdivision
1	Sand	S, Su2
2	Loamy sand	St2, Sl2, Sl3, Su3, Su4, Us, Uu
	Sandy silt	
3	High sandy loam	Sl4, Slu, Uls, Ut2, Ut3
	Loamy silt	
4	Sandy loam	St3, Ts4, Ls4, Lts, Lt2, Ls3, Ls2, Lu, Ut4, Ts3
	Silty loam	
	Loam	
5	Silty clay loam	Tu3, Lt3, Tu2, Tl, Ts2, Tt, Tu4
	Clay loam	
	Clay	

Table S 5: *Microwave-assisted hydrofluoric acid (HF) digestion of German soils.*

Microwave program	Power [W]	Ramp [min]	Hold time [min]	Van
I	1400	5	35	1
	0	–	20	3
II	950	5	15	1
	0	–	15	3

A Microwave 3000 from Anton Paar combined with HF-resistant Teflon® microwave vessels were used for HF digestion of German soil samples. 0.5 g of air-dried and grounded soil samples were filled in the vessel and mixed with the following acids: 5 mL 65 % HNO_3 (sub-boiled), 2 mL (32 %) HCl (sub-boiled) and 3 mL 32 % HF (for analysis). The vessels were closed and microwave program I was conducted. After digestion 20 mL of saturated H_3BO_3 was added and microwave program II was started. The solution was cooled down to room temperature, filtered, transferred into a 50 mL volumetric flask and filled up with ultrapure water. From each sample two digests were made.

Table S 6: *Specification of the ICP-OES set-up used for analysing the German soil samples.*

Specification	5110
company	Agilent
software	ICP-Expert
spectrometer	Echelle polychromator
detector	VistaChip II CCD
plasma viewing	dual (axial, radial)
spray chamber	ICP double pass inert ball
nebulizer	OneNeb
power [W]	1200
plasma gas [L/min]	12
auxiliary gas [L/min]	1
nebulizer gas [L/min]	0.7
autosampler	Agilent SPS 3
wavelength [nm]	K(I) 766.491, K(I) 769.897, Ca(II) 317.933, Ca(II) 393.366, Ca(II) 422.673, Fe(II) 238.204, Fe(II) 259.940, Fe(II) 234.350

Table S 7: *Main operating parameters of WDXRF MagiX Pro for detection of K, Ca and Fe* K_α.

**Specification**	**MagiX Pro**
company	Panalytical
set-up	wavelength-dispersive
software	SuperQ Version 5.0B
tube	Rh-anode
medium	He
max. power	4 kW

Measuring parameter

**Element**	**Crystal**	**Collimator [µm]**	**Detector**	**Voltage [kV]**	**Current [mA]**	**Angle [°2T]**
K	LiF 200	150	Flow	32	125	136.72
Ca	LiF 200	150	Flow	32	125	113.14
Fe	LiF 200	150	Flow	60	66	57.53

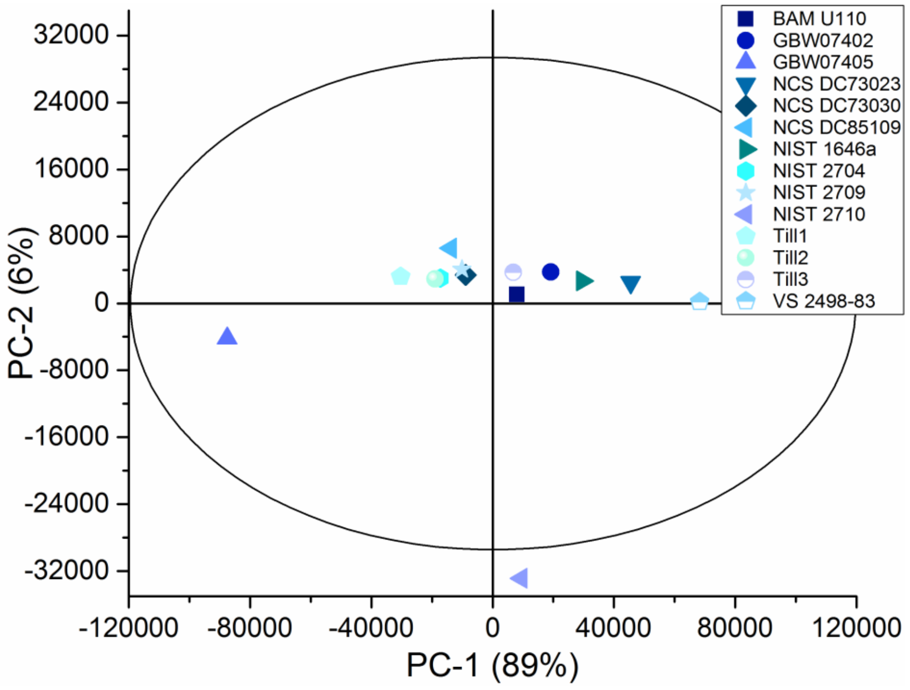

Figure S 1: *PCA score plot of EDXRF soil data for the first two principal components PC-1 and PC-2 of 14 certified reference materials with a total variance of 95 %. NIST 2710 is located beyond the T² ellipse.*

Explanation: As shown in Figure S 1 CRM NIST 2710 is located beyond the T² ellipse and was therefore excluded from the PCA. Because the PCA did not describe this CRM quite well. CRM NCS DC 87104 was also excluded. The certified value for Ca was significantly underestimated with the EDXRF. Since PCA takes the CRM as a basis to classify the real soils and the second PC belongs to Ca, this CRM was not used for modelling the PCA.

Table S 8: *Samples used for potassium calibration uni- and multivariate with a selection of German soil samples.*

study site	sample	reference mass content WDXRF [wt-%]	amount of samples
Marquardt	A1	1.539	1
An der Chaussee, Gut Wilmersdorf	ATB	1.967; 2.084; 2.301	3
Kohlgarten, Gut Wilmersdorf	ATB	1.225; 1.403	2
Rotes Meer, Gut Wilmersdorf	ATB	1.422; 1.533	2
Waschpuhl, Gut Wilmersdorf	ATB	2.132; 2.247; 2.279	3
Campus Klein-Altendorf, Bonn	G3	1.894	1
Goerzig	Goerzig	1.617; 1.682; 1.697; 1.721; 1.780; 1.825; 1.917; 2.032; 2.339	9
Zalf, Muencheberg	Zalf	1.543; 1.702; 1.812; 2.142	4

Grafschaft Boelingen	UBR	0.740; 0.871; 0.919; 1.216; 1.321; 1.689; 1.733; 1.776; 1.853	9
Various soil samples with unknown site	Tex, UB	1.160; 1.044; 1.447; 2.501; 2.922; 3.021	6
Speyer	Lufa	1.921	1

Table S 9: *Samples used for iron calibration uni- and multivariate with a selection of German soil samples.*

**study site**	**sample**	**reference mass content WDXRF [wt-%]**	**amount of samples**
Marquardt	A	0.597; 0.718	2
An der Chaussee, Gut Wilmersdorf	ATB	1.092; 1.466; 1.735; 2.662	4
Kohlgarten, Gut Wilmersdorf	ATB	0.826; 2.343	2
Rotes Meer, Gut Wilmersdorf	ATB	0.833; 1.005; 1.025	3
Waschpuhl, Gut Wilmersdorf	ATB	2.577; 2.671; 2.808	3
Ascheberg, Bonn	G	1.286; 2.779	2
Campus Klein-Altendorf, Bonn	G	1.932; 2.209	2
Goerzig	Goerzig	1.86; 1.992; 2.031; 2.107; 2.158; 2.325; 2.656; 2.668	8
Zalf, Muencheberg	Zalf	0.740; 0.851; 0.961; 1.031	4
Grafschaft Boelingen	UBR	3.127; 3.666; 4.467; 5.114; 5.163	5
Various soil samples with unknown site	Tex, UB	1.346; 1.456; 2.748; 3.317; 4.692; 5.015; 5.754; 5.827	8
Speyer	Lufa	1.131	1

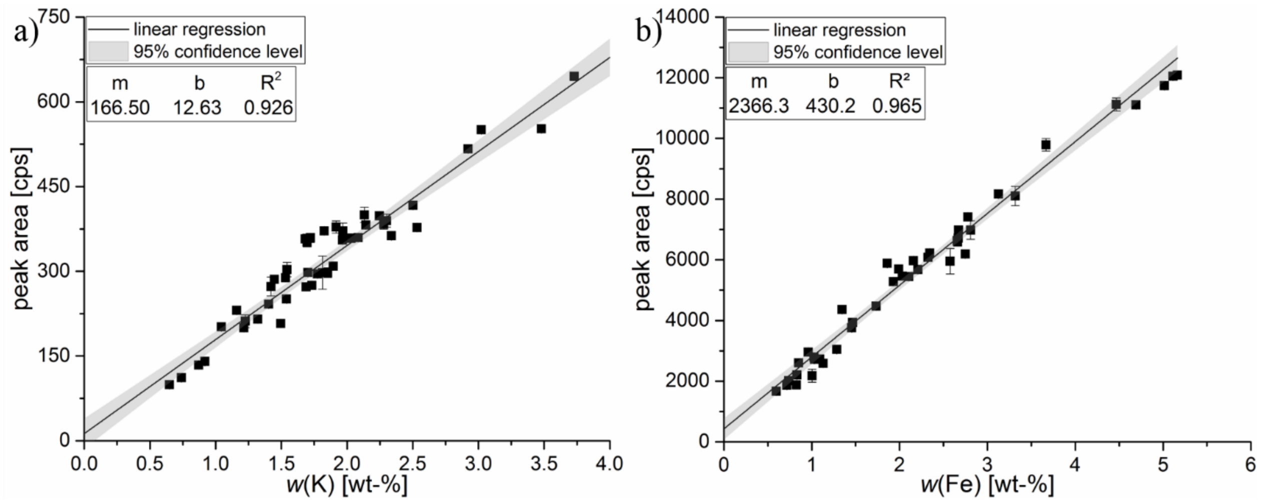

Figure S 2: *Univariate calibration model for potassium (a) and iron (b) based on a selection of German agricultural soils. The net peak area of the characteristic fluorescence peak [cps] was fitted against the mass fraction in the German soils [wt-%] determined by WDXRF. The error bars represent the standard deviation of five measurements.*

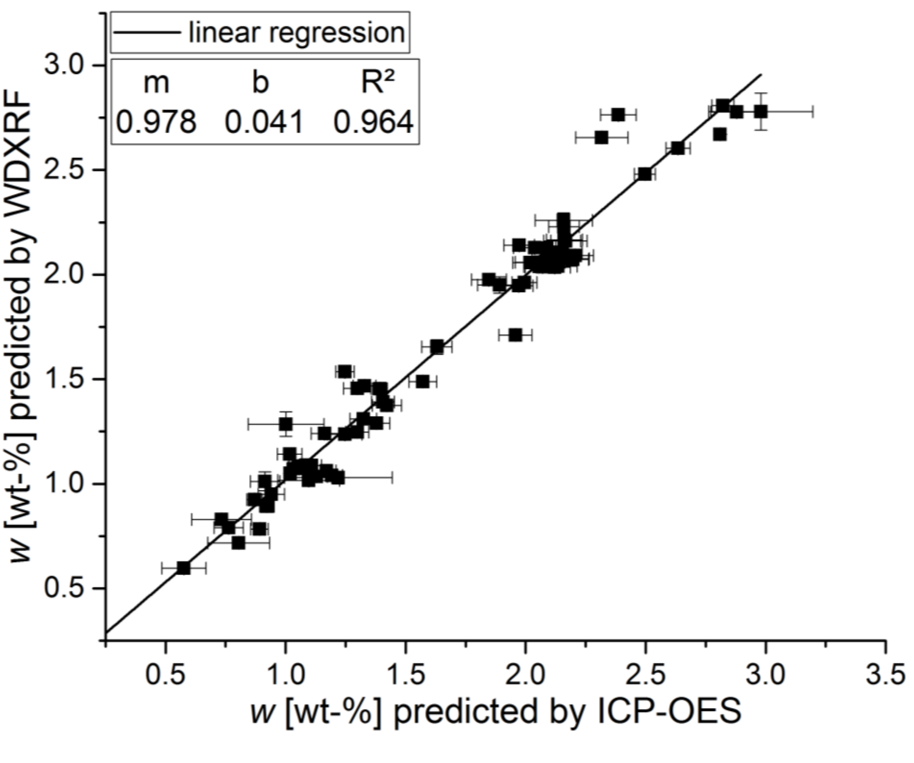

Figure S 3: *Reference values of WDXRF plotted against the reference values for iron in German soils determined by ICP-OES with 95% confidence level. The error bars represent the standard deviation of two WDXRF and two ICP-OES measurements.*

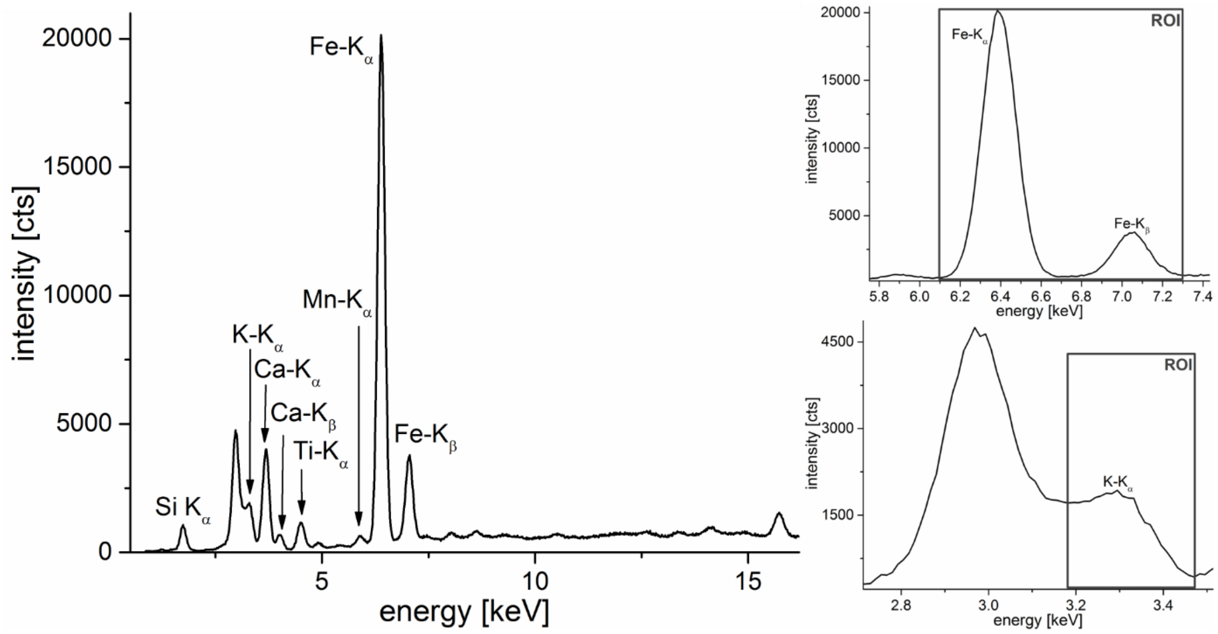

Figure S 4: *Representative EDXRF spectrum of a soil sample from arable land in the region of interest (ROI) with important peaks marked. The fluorescence intensity [cts] is plotted against the element characteristic energy [keV]. Acquisition time: 60 s, Voltage: 29.7 kV, Current: 0.49 mA.*
